# Accuracy and Safety of Late Chorionic Villus Sampling in High-Risk Pregnancies in 8599 Cases

**DOI:** 10.3390/genes16080860

**Published:** 2025-07-24

**Authors:** Petra Podobnik, Mario Podobnik, Ivan Bertovic-Zunec, Igor Lončar, Kristijan Kurdija, Dženis Jelčić, Zlata Srebrenikovic

**Affiliations:** Department of Obstetrics and Gynecology, Sveti Duh 112, 10 000 Zagreb, Croatia; petra@podobnik.hr (P.P.); bertovc.zunec.ivan@podobnik.hr (I.B.-Z.); igor.loncar@podobnik.hr (I.L.); kristijan.kurdija@podbnik.hr (K.K.); dzenis.jelcic@podobnik.hr (D.J.); srebrenikovic.zlata@podobnik.hr (Z.S.)

**Keywords:** late placental biopsy (LCVS), spontaneous abortion, suspicious ultrasonographic findings, chromosomal abnormalities, aneuploidies

## Abstract

Objectives: To evaluate the association between late CVS (placental biopsy, later than 13 weeks of gestations) and complications between sampling and delivery in 8599 cases in the Department of Obstetrics and Gynecology of a private hospital Podobnik, Zagreb, Croatia. Methods: Late chorionic villus sampling under ultrasound guidance was carried out in prospective monocentric cohort study of 7859 (91.4%) cases in the second trimester and 700 (8.6%) cases in the third trimester of pregnancy. Out of 8599 late CVS cases, 1476 (17.2%) were performed because of suspicious ultrasonographic findings. Results: In 43 patients (0.50%), complications were found between sampling and delivery. There were only 12 (0.15%) spontaneous abortions four to six weeks after late CVS (before 28 weeks). We found 190 (2.3%) chromosomal abnormalities. In the group with suspicious ultrasonographic findings, comparing 1476 cases, we found significant oligohydramnios in 375 (25.4%), significant polyhydramnios in 197 (13.3%) and chromosomal abnormalities in 125 (8.5%) cases. Among the 190 patients with chromosomal abnormalities, ultrasonographic findings were detected in 98 (49.2%) after the 22th week of pregnancy. Conclusions: Late CVS is a safe method of invasive prenatal diagnosis with lower spontaneous abortions rate (0.15%). This method, applicable after 13 weeks of gestation, offers a more flexible approach to invasive prenatal diagnosis of chromosome abnormalities, in very specialized fetal-maternal centres for this method.

## 1. Introduction

The pioneering work of Alvarez [1] demonstrated the possibility of obtaining transabdominal villus sampling from an in situ placenta. Subsequently a variety of methods have been described to villi samples. Hodgal et al. [2] made significant contributions to the feasibility of conducting chromosome studies on the trophoblast. After the reports by Chieri and Aldini [3], Hodgal et al. [2] and Nicolaides et al. [4], we began to offer late chorionic villus sampling to the patients who were late for early chorionic villus sampling and some of those regularly scheduled for amniocentesis to accept the cytogenetic results for three weeks. A rapid karyotype result is of importance when patients who are at an increased risk for fetal chromosome disorders present for prenatal diagnosis near 22 weeks gestation. Similarly, patients who present late in the second trimester with abnormal ultrasound findings or oligohydramnios can be considered candidates for second-trimester chorionic villus sampling due to an increased risk of fetal chromosome abnormalities. The findings may lead to elective termination of pregnancy or in some circumstances vaginal delivery, rather than cesarean The primary aim of this prospective monocentric cohort study was to evaluate the association between invasive prenatal diagnostic procedures and very low spontaneous abortions rate after late CVS. An additional objective was to describe the clinical outcomes of affected pregnancies over a 15-year period at tertiary care centres in the Republic of Croatia.

## 2. Patients and Methods

**Figure 1:**
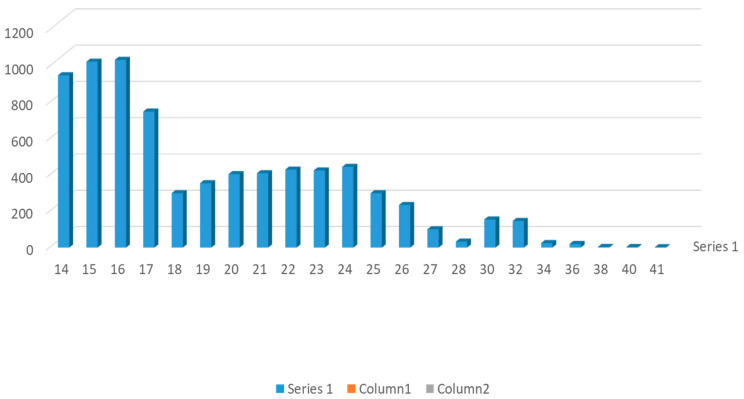
Number of procedures per week in 8599 patients.

Between January 2008 and May 2023, late chorionic villus sampling was performed in a prospective, monocentric, cohort study on 8599 singleton pregnancies from 14–41 weeks of pregnancy. A total of 7799 cases were between 14–28 weeks of pregnancy, and 700 cases were between 29–41 weeks of pregnancy (Figure 1). The indications for tissue sampling are in Table 1. Exclusion criteria involved the detection of embryo death at the first ultrasound, multiple pregnancies, or if they were RH-isoimunized, or untreated cervical infection and cases were offered placental biopsy or amniocentesis for private reasons. Gestational age as determined by the last menstrual period and early ultrasound measurement. Demographic and other perinatal information including obstetrical and genetic history, was collected and extracted from electronic records with standardized reports. The maternal age at sampling from 18–52 years, with 77% of patients over 37. Before sampling, all women underwent accurate clinical and ultrasonic evaluation. When the late CVS was in the second trimester of pregnancy, all women were offered a follow-up ultrasound examination one week after sampling, and again between 16 and 20–22 weeks and 26–28 weeks, when completed and returned the questionnaire about the progress of their pregnancies. When the sampling was in the third trimester of pregnancy, all women were offered a follow-up ultrasound examination one week after sampling and again between 32 and 34 weeks, at 36 weeks and before delivery. The delivery hospitals identified any complications that occurred during pregnancy and delivery, as well as information about the newborn and the placenta. The follow-up of all 8799 patients was completed. Given the variability in outcomes, the management of pregnancies complicated after late CVS should be multidisciplinary, involving fetal medicine specialists, genetic counselors and neonatologists.

The maternal abdomen was prepared with a topical of povidone-iodine, and a sterile drape was placed over it. No local anesthetic was given. Sampling was performed under real-time ultrasonographic guidance using a curvilinear transducer with a frequency of 2.5–5 MHz, protected with a sterile plastic bag. A 20 or 18-gauge spinal needle was introduced through the abdominal maternal wall to the placenta under continuous ultrasound guidance inserted vertically and sometimes obliquely in the placenta. We used a 20-gauge single spinal needle tip for sampling from week 14 to 16 and an 18-gauge needle after week 16. When the echo of the needle was within the placenta, the stylet was removed with a slow movement in the presence of negative pressure, placental tissue was aspirated into a 20 mL syringe containing of culture medium Furthermore, all rhesus-negative women received 50 μg of anti-D administrated after sampling.

Cytogenetic findings [1,2,3,4,5,6,7,8,9,10,11,12,13,14,15,16] indicated that the sample of villus tissue was immediately transferred a laboratory and examined under a dissecting microscope. An overnight short-term incubation method for three days or a culture method for seven days was used [7,8,11,12,13,14,15,16]. Additionally, fetal heart rate (FHR) was monitored by M-mode in all patients.

Doppler recordings of the uterine artery, the spiral artery, the chorionic artery, the intraplacental arteriole (near the sample place), the umbilical artery, the the ductus venosus, and the middle cerebral artery were performed 10 min prior to sampling and 10 min after procedures in 1300 patients between 14 and 20 weeks [7,8,9,13,14,15,16,17,18,19,20]. Doppler recordings were performed in the same group of patients in which the concentration of maternal serum AFP was measured to evaluate possible fetal vascular damage [6,7,15,16,17,18,19,20]. The study was performed using a GE machine (Voluson E8 and Voluson 10) with a transabdominal probe of 2–5 MHz Once the anatomical position of the investigated area obtained the pulsed wave Doppler sample volume was focused on the structure of interest. The pulsatility index (PI) was calculated automatically, and the mean value was determined. The statistical analysis was carried out using the Student’s *t*-test and chi-square test. To evaluate statistical significance where applicable, a *p*-value of <0.05 (two-tailed) was considered statistically significant. Ethical approval was granted by the Ethics Committee of the Podobnik Department of Obstetrics and Gynecology of Zagreb (13 August 2008, No 1–13). Informed consent was obtained from all subjects involved in the study.

## 3. Results

In 70% of patients the placenta was anterior or fundal, and the success rate of obtaining sample was 99% after the first sampling and 100% after the second sampling. In 30% of patients, the placenta was posterior, and the success rate of obtaining was 98% after the first sampling and 100% after the second sampling. In 35 (0.4%) patients, we found early complications between sampling and delivery (Table 2). Lower back pain and uterine contraction were found in 12 (0.15%) cases in the second trimester of pregnancy and 3 (0.4%) cases in the third trimester of pregnancy. Fever was found in three (0.3%) cases, but no instances of chorioamnionitis developed. There were only 12 (0.15%) spontaneous abortions after late chorionic villus sampling, four to six weeks later. All women were older than 37 years, chorionic villus sampling was carried out in the 14th to 18th weeks and spontaneous abortions after sampling were carried out in the 17th and 24th weeks. Another six women had late chorionic villus sampling after 18th weeks and spontaneous abortions in the 24th week (karyotype was trisomy 21 in four and trisomy 18 in two women, but the parents declined elective abortion). In all 12 women the amount of tissue was between 30 and 50 mg.

The concentration of total mean AFP) levels increased after sampling in 25 (0.5%) patients from 500 patients in whom serum samples were obtained before and after sampling. Ten of them were spontaneous abortions after sampling, and all ten had chromosomal aberrations. However, no correlation was found between AFP elevation, placental hematoma and fever after sampling.

Cytogenetic findings were obtained in 8599 (99.9%) placental samples. In 10 cases, metaphases were unsuitable for study. The over-night direct method was utilized in 60.0%, culture in 20.9%, and culture and over-night technique in 19.1% of the same group of patients (Table 3). We found chromosomal aberration in 190 (2.2%): trisomy 21 (68 cases), trisomy 18 (35 cases), trisomy 16 (6 cases), trisomy 13 (33 cases), monosomy X (27 cases), 46XXY (21 cases) and in 25 (0.3%) patient mosaicisms were found. These mosaicisms were confirmed (in 15 cases) or not confirmed (in 10 cases) in fetal blood sampling by cordocentesis. The total fetal loss in all cases was 150 (1.8%), in 57 (8.1%) women in whom late chorionic villus sampling was performed in the second trimester of pregnancy and 5 (0.5%) in women in whom late chorionic villus sampling was performed in the third trimester of pregnancy. In the group with suspicious ultrasonographic findings, comprising 1476 cases, we found significant oligohydramnios in 375 (25.4%), significant polyhydramnios in 197 (13.3%) and chromosomal abnormalities in 125 (8.5%) cases (Table 4). Among the 60 patients with chromosomal abnormalities, ultrasonographic findings were detected in 10 (16.7%) after the twentieth week of pregnancy. In all 10 cases, the obstetric management was changed of abnormal cytogenetic results and abnormal ultrasonographic findings. Five of those had an elective abortion, three fetuses with ultrasonographic abnormalities and oligohydramnios detected between 28 and 32 weeks were stillborn, and two had an early neonatal death. The concurrent fetal abnormalities detected in this study expectedly resulted in a higher proportion of total fetal loss. We did not see any serious limb abnormalities after late chorionic villus sampling.

Color Doppler was used to investigate the uteroplacental and fetal vessels in 1300 (30.0%) pregnancies between 14 and 20 weeks of gestation (Table 5). The main uterine artery, the spiral arteries, the umbilical artery, the chorionic artery, the intraplacental arteriole (near the sample place), the ductus venosus, and the middle cerebral artery were investigated. There were no significant differences in mean pulsatility indices (PISs) between maternal and fetal circulation before and after sampling. The preliminary data of 50 trisomic fetuses (forty trisomy 21, five trisomy 18 and five trisomy 13) have abnormally increased umbilical PIs in the umbilical artery and ductus venosus and abnormally decreased PIs in the middle cerebral artery (Table 6).

## 4. Discussion

In our study there were only 12 (0.15%) spontaneous abortions after late CVS sampling. There were no spontaneous abortions after sampling in the latest 1000 pregnancies with cytogenetically and sonographically normal fetuses, to allow meaningful conclusions about the safety of the procedure. In comparison Holzgreve et al. [8,9] report a loss rate of 2.3% in 728 pregnancies from 24 centres after late CVS sampling. Smidt-Jensen et al. [21] reported one (0.7%) fetal loss in a group of 139 sonographically and cytogenetically normal fetuses 20 weeks after sampling. In our group the spontaneous abortion rate after late CVS sampling is low and compatible with mh Smit-Jensen et al. and Jahoda et al. [22] studies. Our study showed a decrease in the fetal loss rate with advancing gestational age and the relationship between fetal loss and maternal age. In younger women this rate was not influenced by gestational age. Holzgreve et al. [8,9] found as many as 20% of chromosomal aberrations in cases with sonographic abnormalities. Chromosomal aberrations have been seen in as many as 32% of fetuses with an abnormal fetal echography, compatible with 33.3% in our series. Among the 60 patients with suspicious ultrasound findings or intrauterine growth retardation detected after the twenty weeks of pregnancy, 10 (16.7%) fetuses had chromosomal abnormalities. We did not find a correlation between AFP elevation, placental hematoma, Doppler measurements, and spontaneous abortion rate after late chorionic villus sampling. Our results are in agreement with previous studies in which a marked rise in AFP after early and late CVS was not associated with fetal demise. The risk of spontaneous abortion after the second trimester transabdominal chorionic villus sampling is 0.4% to 3.2% [5,6,7,8,9,10,12,16,17,21,23,24,25,26,27,28,29,30,31,32,33,34]. In our study, there were only 12 (0.15%) spontaneous abortion after sampling. Chromosomal aberrations have been seen in as many as 32% of fetuses with an abnormal fetal echography, compatible with 33.3% in our series. Among the 60 patients with suspicious ultrasound findings or intrauterine growth retardation detected after the twenty weeks of pregnancy, 10 (16.7%) fetuses had chromosomal abnormalities. In all 10 of these cases, the obstetric management was changed because of abnormal cytogenetic results. The primary aim of this prospective monocentric cohort study was to evaluate the association between invasive prenatal diagnostic procedures (late CVS) and very lower spontaneous abortions rate after late CVS, in our cases in 0.15%, The problem while late CVS don’t change classical amniocentesis after 15 weeks of gestations, were in cytogenetic laboratories which done not briefly short technique for three days or culture methods for seven days. That is the reason why our study was larger in the world. On this day there were only 15.000 late CVS, and we had lower complications after late CVS procedures in 8599 cases.

In our laboratory, more than 5 mg of placental tissue was needed to perform the cytogenetic studies [12,18,22,27,28,29,35,36], The mitotic index on placental villi decreased with increasing gestational age: Saura et al. [5] reported a result at 35 weeks, Nicolaides et al. [4] reported results at 37 weeks and in our hands 41 weeks gestation seems to be limited, to approximately one metaphase on the 500 cells [12,16]. Many cytogenetic laboratories need approximately 30–50 mg of placental tissue and this massive sampling is a reason way had spontaneous abortion after late CVS in 1–3.2% patients. That was a reason why late CVS don’t change classical amniocentesis after 15 weeks of gestation. Mosaicisms are considered to occur with a frequency of approximately 1–2%, with confirmation not typically occurring in most patients [3,4,6,7,10,12,16,17,24,25,27,28,29,30,31,32,33,34,36,37,38,39,40,41]. In our study we found mosaicisms in 0.3% of cases. These mosaicisms were confirmed (in 15 cases) or not confirmed (in 10 cases from fetal blood sampling by cordocentesis [12,18,21,22,27,28,35,36,42].

We did not find a correlation between AFP elevation, placental hematoma, Doppler measurements and spontaneous abortion late chorionic villus sampling. Therefore, the amount of fetomaternal bleeding in the second and third trimester may be in the same range as in the first trimester. Our results are in agreement with previous studies in which a marked rise in AFP after early and late CVS was not associated with fetal demise [12,20,21,25,27,28,29,30,36].

To evaluate possible fetal vascular damage, we assessed fetal and maternal circulation using color Doppler and measured AFP in maternal serum. There were no significant differences in mean pulsatility indices between maternal and fetal circulation before and after late chorionic villus sampling. This suggested that uteroplacental and fetal blood flow were also not affected by placental biopsy procedures and the subsequent fetal loss rate was not the result of pathological alterations in uteroplacental and fetal vasculature. Given the variability in outcomes, the management of pregnancies complicated by fetal structural abnormalities should be multidisciplinary, involving fetal medicine specialists, genetic counsellors and neonatologists.

Late CVS applicable after 13 weeks of gestation, offers a more flexible approach to invasive prenatal diagnosis of chromosome abnormalities, in very specialized fetal-maternal centres for this method.

## Figures and Tables

**Table 1:** Indication for late CVS in the second and third trimesters of 8599 patients.

Indication	N	Normal Karyotype	Chromosomal Aberration
Maternal age of 37 or higher	5589	5551	38
Failed amniocentesis	220	217	3
Prior chromosome anomaly	285	265	25
Susp. ultrasound findings of chromosomal aberration or susp. NIPTI testes	825	740	85
Other suspicious ultrasound malformations	395	369	26
Anxiety	50	48	2
Others	55	54	1
Total	8599	8424	190 (2.2%)

Mosaicisms were present in 25 cases (0.3%).

**Table 2:** Pregnancy outcome after late CVS in 8599 patients.

In the Second Trimester (14–28 Weeks) N = 7899 (91.9%)			In the Third Trimester (29–41 Weeks) N = 700 (8.1%)	
Early Complication	N	%	N	%
Lower back pain and uterine contraction	12	0.2	5	1.0
Placental hematoma	8	0.1	3	0.3
Infection	3	0.1	1	0.1
Spontaneous abortion	12	0.15	0	0
Chromosomal abnormalities	134	1.7	41	5.9
Delivered < 37 weeks	389	4.5	50	7.1
Delivered >37 weeks	7510	95.5	650	92.9
Stillbirth	9	0.1	10	1.2
Total	143	3.5	51	7.2
Women > 37 years	94	65.7	40	78.4
Women < 37 years	49	34.3	11	31.6

**Table 3:** Cytogenetic techniques performed.

Cytogenetic Technique	N	%
Overnight technique	5149	60
Culture (seven days)	1799	20.9
Culture and overnight technique	1641	19.1
Total	8599	

**Table 4:** Cytogenetic aberration found in pregnancies with suspicious ultrasound findings.

Ultrasound Findings	N	Chromosomal Aberations	N
Susp.ultrasound findings of fetal aneuploidies	811	Trisomy 21 Trisomy 18 Trisomy 13	32 4 20
Oligohydramnios with or without IUGR	374	Trisomy 21 Trisomy18 Trisomy 13	16 12 5
Polyhydramnios	175	Trisomy 21 Trisomy 18 Trisomy13	5 5 2
Hydrocephalus	10	Trisomy 21 Trisomy 18 Trisomy 13	3 3 2
Hygroma colli	10	Trisomy 21 Monosomy X	2 8
Cardiac anomaly	30	Trisomy 21 Trisomy 18 Trisomy 13	9 4 2
Thotracoschisis	4	Trisomy 18	2
Omphalocele	10	Trisomy 18 Trisomy 13	5 2
Hydronephrosis and dysplastic kidneys	10	Trisomy 21 Monosomy X Trisomy 16	4 3 3
Other ultrasound abnormalities	21	Trisomy 21 Trisomy 16 Monosomy X 47 XXY	2 3 16 21
Total Number	1476 (17.2%)	Chromosomal Abberations	190 (12.9%)

**Table 5:** Pulsatility indices (PIs) values for maternal and fetal circulation before and after CVS.

N = 1300	PI 10 min	Before PB	PI 10 min	After PB	*p*
	Mean	SD	Mean	SD	
Uterine ar.	1.17	0.43	1.01	0.30	>0.05
Spiral ar.	0.44	0.02	9.41	0.03	>0.05
Intraplacental arteriole	0.55	0.02	0.52	0.03	>0.05
Chorionic ar.	0.88	0.05	0.85	0.04	>0.05
Umbilical ar.	1.69	1.02	1.66	1.01	>0.05
Fetal aorta	1.70	0.45	1.60	0.40	>0.05
	PI=	Pulsality Index	PB	Placental Biopsy	

**Table 6:** Pulsatility indices (PIs) values in maternal and fetal circulation before and after late CVS in fetuses with chromosome abnormalities.

N = 175	PI 10 min	Before PB	PI 10 min	After PB	*p*
	MEAN	SD	MEAN	SD	
Uterine ar.	1.17	0.43	1.01	0.30	>0.05
Spiral ar.	0.44	0.02	9.41	0.03	>0.05
Intraplacental arteriole	0.55	0.02	0.52	0.03	>0.05
Chorionic ar.	0.88	0.05	0.85	0.04	>0.05
Fetal aorta	1.70	0.45	1.60	0.40	>0.05
Umbilical ar.	1.68	1.01	2.20	0.60	<0.05, t = 2.12 *
Middle cerebri artery	2.01	0.45	1.50	0.50	<0.05, t = 6.92 *
Ductus venosus	0.90	0.30	3.30	0.45	<0.05, t = 37.3 *
	PI=	Pulsality Index	PB=	Placental Biopsy	

* N = 50 aneuploidic fetuses had significant difference.

## Data Availability

The data presented in this study are available from the corresponding author on request.
